# In Situ Hydrogel Formulation for Advanced Wound Dressing: Influence of Co-Solvents and Functional Excipient on Tailored Alginate–Pectin–Chitosan Blend Gelation Kinetics, Adhesiveness, and Performance

**DOI:** 10.3390/gels10010003

**Published:** 2023-12-20

**Authors:** Chiara Amante, Giovanni Falcone, Rita P. Aquino, Paola Russo, Luigi Nicolais, Pasquale Del Gaudio

**Affiliations:** 1 Department of Pharmacy, University of Salerno, Via Giovanni Paolo II, 132, 84084 Fisciano, Italygifalcone@unisa.it (G.F.); aquinorp@unisa.it (R.P.A.); paorusso@unisa.it (P.R.); 2 Materias s.r.l., University of Naples “Federico II” Campus San Giovanni a Teduccio, 84146 Naples, Italy; 3 Research Centre for Biomaterials BIONAM, University of Salerno, Via Giovanni Paolo II, 132, 84084 Fisciano, Italy

**Keywords:** in situ gelling powder, wound dressing, alginate, pectin, chitosan

## Abstract

Chronic skin wounds affect more than 40 million patients worldwide, representing a huge problem for healthcare systems. This study elucidates the optimization of an in situ gelling polymer blend powder for biomedical applications through the use of co-solvents and functional excipients, underlining the possibility of tailoring microparticulate powder properties to generate, in situ, hydrogels with advanced properties that are able to improve wound management and patient well-being. The blend was composed of alginate, pectin, and chitosan (APC). Various co-solvents (ethanol, isopropanol, and acetone), and salt excipients (sodium bicarbonate and ammonium carbonate) were used to modulate the gelation kinetics, rheology, adhesiveness, and water vapor transmission rate of the gels. The use of co-solvents significantly influenced particle size (mean diameter ranging from 2.91 to 5.05 µm), depending on the solvent removal rate. Hydrogels obtained using ethanol were able to absorb over 15 times their weight in simulated wound fluid within just 5 min, whereas when sodium bicarbonate was used, complete gelation was achieved in less than 30 s. Such improvement was related to the internal microporous network typical of the particle matrix obtained with the use of co-solvents, whereas sodium bicarbonate was able to promote the formation of allowed particles. Specific formulations demonstrated an optimal water vapor transmission rate, enhanced viscoelastic properties, gel stiffness, and adhesiveness (7.7 to 9.9 kPa), facilitating an atraumatic removal post-use with minimized risk of unintended removal. Microscopic analysis unveiled that porous inner structures were influencing fluid uptake, gel formation, and transpiration. In summary, this study provided valuable insights for optimizing tailored APC hydrogels as advanced wound dressings for chronic wounds, including vascular ulcers, pressure ulcers, and partial and full-thickness wounds, characterized by a high production of exudate.

## 1. Introduction

Wound healing is a vital biological process that restores the integrity of the skin and other tissues after injury or disease. It is an intricate and dynamic biological phenomenon, comprising four interrelated phases: hemostasis, inflammation, proliferation, and remodeling, which are regulated by various cell types, growth factors, chemokines, and cytokines [1,2,3]. However, in the case of chronic wounds, which affect more than 40 million patients globally, the normal healing process is severely impaired or delayed, representing a severe and growing burden on the healthcare system [4]. Therefore, the goal of wound management is to heal the wound using a specific wound dressing that leads to the closure of the wound [5], reducing, at the same time, the risk of infections and minimizing the discomfort of the patient. Conventional wound dressings, such as gauze and those based on the principle of mere “covering and concealing”, have several limitations, such as obstructing oxygen diffusion, creating a dry environment, and causing pain and trauma during removal [6,7,8]. Therefore, there is a need for more advanced wound dressings that can provide a moist, breathable, and antimicrobial barrier, as well as deliver therapeutic agents to the wound site. In the last few years, several innovative wound dressings have been studied; among them, hydrogels have the most promising features [9]. In fact, hydrogels exhibit multifarious virtues, including microbial barrier properties, facilitation of gaseous exchange, and the potential for controlled drug release [10,11,12]. Noteworthy for their conformability, hydrogels can be applied and removed painlessly, while their transparency allows for monitoring of the healing process [13]. The choice of dressing must also consider the nature of the wound, as well as the exudate management [7,14]. Exudate from acute wounds contains proteins vital for epithelial cell nourishment, alongside a balanced array of proteolytic enzymes and their inhibitors, priming the wound bed for closure and remodeling [15]. In contrast, exudate from chronic wounds is rich in proteinases that block the proliferation of vital cells pivotal for the wound-healing process [16]. Furthermore, chronic wounds, such as venous leg ulcers, diabetic wounds, and pressure ulcers, present an intensified risk of the development of drug-resistant bacteria and persistent infections [17,18].

Among the different innovative materials used for the production of hydrogel for wound healing, natural polymers such as alginate [19], fibrin [20], chitosan [21,22], pectin, and gelatin [23] have gained much attention due to their biocompatibility and biodegradability [23,24].

Alginate, a polysaccharide derived from algae, is composed of mannuronic (M) and guluronic (G) acid units [25]. It is particularly useful in wound dressing due to its ability to absorb exudate and form hydrogel [26,27]. Moreover, alginate with a high M content induces the release of cytokines involved in wound healing at a higher rate than alginate with a high G content [28,29].

Pectin, a water-soluble polysaccharide, easily obtained by extraction from fruits and vegetables, has recently been explored regarding its use in numerous biomedical applications, including drug delivery [30]. However, its intrinsic mechanical fragility requires a combination with other polymers [31].

Chitosan, composed of D-acetylglucosamine and D-glucosamine, is a biopolymer widely used in a variety of biomedical fields [32,33]. The molecular weight of chitosan can influence its physicochemical properties, such as viscosity, solubility, and elasticity [34]. Furthermore, regardless of molecular weight, chitosan can promote wound healing thanks to anti-inflammatory, homeostatic, and antimicrobial activity [35,36,37].

Natural polymers, when properly mixed, can produce composites showing enhanced properties compared to their constituents [38,39]. In a previous work [40], we showed the potential of exploiting spray drying to obtain a polymeric powder blend of alginate, pectin, and chitosan (APC) able to form a hydrogel in situ that can be used as a wound dressing. Fluid uptake demonstrated that all APC formulations were able to reach the maximum uptake in about 10 min. With the aim to improve the technological properties of this powder, such as the in situ gelling process, the pivotal attribute for wound-healing application, the investigation of this work has covered the impact of several co-solvents (ethanol, isopropanol, and acetone) or additional excipients in the form of salts, like sodium bicarbonate and ammonium carbonate on the powder production. The investigation assessed the gelation kinetics, rheological properties, and water vapor transmission rate of the ensuing in situ hydrogel.

## 2. Results and Discussion

Alginate–pectin–chitosan (APC) polymeric particle blends were produced using mini spray drying, setting a total polymer concentration of 0.15% (*w*/*v*). In a previous work [40], the influence of chitosan on the gelling properties of carbohydrate-based powder blends was explored, producing formulations containing a high chitosan content, namely APC 117, which exhibited superior gelling performance. To further enhance both gelling and adhesion properties of the hydrogel generated in situ, the use of several co-solvents in the feed compositions were evaluated. In fact, the addition of more volatile solvents into the aqueous spray drying feed can potentially modify the drying mechanism, thereby influencing crystallization kinetics and the characteristics of the resultant particles [41,42]. Compared to water, organic co-solvents can speed up solvent evaporation and promote the formation of hollow or porous particles [43,44].

Feeds incorporating 5%, 10%, and 20% (*v*/*v*) of ethanol, 20% (*v*/*v*) of ethanol/acetone (1:1), and 5% (*v*/*v*) of isopropanol in the aqueous-based feed were used for the study. The selection of these solvents was guided by their compatibility with water, their use in pharmaceutical processes, and safety. As summarized in Table 1, the process yield decreased upon the inclusion of co-solvents in the feed, particularly evident with 20% ethanol content, when yield decreased from 61% to 45%. The particle size of powders generated using ethanol as a co-solvent exhibited mean diameters ranging from 3.65 to 5.05 µm, whereas the presence of acetone and isopropanol resulted in reduced diameters, likely attributed to the more rapid solvent removal.

**Figure 1:**
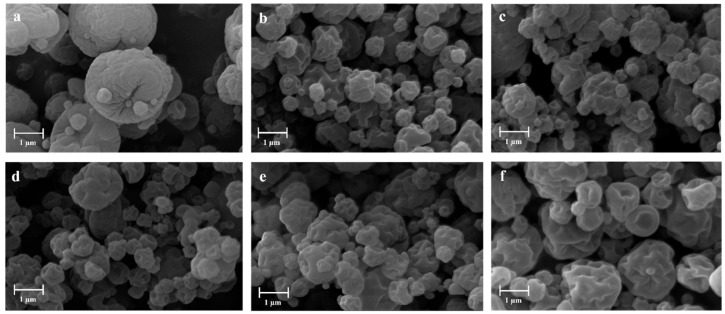
SEM images of powders produced by mini spray drying. (**a**) APC 117, (**b**) APC 117-5Et, (**c**) APC 117-10Et, (**d**) APC 117-20Et, (**e**) APC 117-20EtA, and (**f**) APC 117-5Iso.

Scanning electron microscopy (SEM) showed the distinctive features of the particles with a high content of chitosan particles, such as APC 117, as shown in Figure 1a. These particles showed rough surfaces with pores and clusters, which were probably due to the intricate formation of an alginate–chitosan polyelectrolyte complex that caused aggregation and structural irregularity [45]. The use of a co-solvent affected particle morphology, creating more corrugated particles due to the faster solvent removal (Figure 1b–f). The multi-solvent drying process induced temporal changes in evaporation rates and diffusion coefficients of the solutes within the droplets, known as the Péclet number. The number takes into account both convective and diffusive transport phenomena, providing an idea about the solute behavior in a single-solvent or a multi-solvent system [4]. In a multi-solvent system, like the individual droplets, higher co-solvent content resulted in higher evaporation rates and Péclet numbers, leading to fractured surfaces and low-density hollow particles [46]. Furthermore, the lower solubility of chitosan in ethanol and isopropanol compared to water facilitated the chitosan precipitation after atomization of the water–ethanol feed. This rapid precipitation could cause the collapse of droplet architecture, resulting in the formation of wrinkled particles [47].

To verify the possibility of avoiding precipitation, residual solvent and morphological issues in the particles and the feeds of APC 117 were prepared by the addition of sodium bicarbonate (NaHCO_3_) and ammonium carbonate ((NH_4_)_2_CO_3_) into the aqueous feeds, using concentrations ranging from 2.5 to 10% (*w*/*w*). As reported in Table 1, the addition of sodium bicarbonate to the feed enhanced the process yield and reduced particle size, whereas the inclusion of ammonium carbonate led to both a reduced yield and particle size.

**Figure 2:**
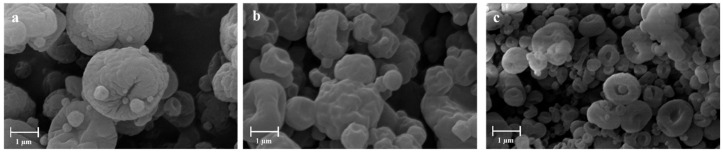
SEM microphotographs. (**a**) APC 117, (**b**) APC 117-10Bic, (**c**) APC 117-10Car.

Moreover, as shown in SEM images (Figure 2), particles made with NaHCO_3_ appeared wrinkled, whereas those obtained with the use of (NH_4_)_2_CO_3_ displayed mainly a discoidal shape and an almost smooth surface.

**Figure 3:**
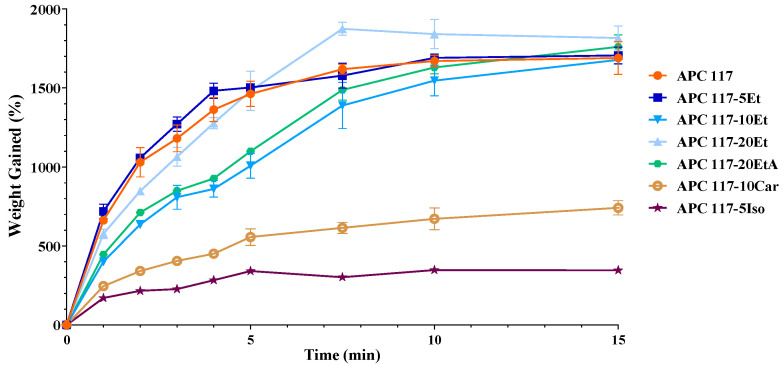
SWF uptake of the hydrogel generated in situ from aqueous feed APC 117 compared to particles obtained by aqueous feeds with co-solvents or addition of (NH_4_)_2_CO_3_: APC 117-5Et, APC 117-10Et, APC 117-20Et, APC 117-20EtA, APC 117-5Iso, and APC 117-10Car.

The powders obtained from different co-solvent-based feeds were used in fluid uptake experiments to assess their ability to absorb simulated wound fluid (SWF). In Figure 3, particles produced with a pure aqueous feed are compared to those produced with aqueous co-solvent feeds. Except for APC 117-5Iso, all formulations exhibited behaviors similar to APC 117, indicating good fluid uptake properties. Notably, APC 117-5Et showed the fastest uptake kinetics, absorbing over 15 times its weight in SWF within 5 min. On the contrary, APC 117-20Et reached the greatest swelling value, but over an extended absorption time. These results suggest that a specific amount of ethanol in the feed might have resulted in particles with different internal structures (porous or open structure), enabling rapid fluid penetration.

Particles produced with the addition of 10% (*w*/*w*) salt in the feeds exhibited an accelerated gelling rate compared to other concentrations. Specifically, powders obtained from the incorporation of NaHCO_3_ were able to completely move to a hydrogel in less than 30 s, difficult to show graphically. On the contrary, when ammonium carbonate was added to the feed, the powders were able to absorb about 10 times their weight in water, moving completely into a gel in 15 min (Figure 3).

**Figure 4:**
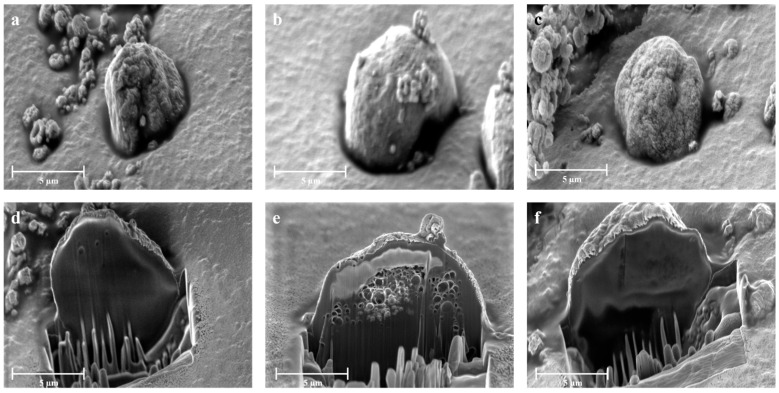
External morphology of (**a**) APC 117, (**b**) APC 117-5Et, and (**c**) APC 117-10Bic obtained by SEM microscopy and cross-section of (**d**) APC 117, (**e**) APC 117-5Et, and (**f**) APC 117-10Bic obtained by FIB-SEM microscopy.

To understand more deeply the inner architecture of the particles, FIB-SEM analyses were conducted, as shown in Figure 4. Through the utilization of a focused ion beam, different internal matrixes were exposed (Figure 4d–f). This outer layer of the particles appears to be made primarily of chitosan, the least soluble of the polymers in the solvent blend, a characteristic more evident with the inclusion of NaHCO_3_ in the aqueous feed. Moreover, APC 117-5Et exhibited an internal matrix characterized by the presence of submicrometric particles, which are the result of the faster evaporation of ethanol. On the other hand, APC 117-10Bic displayed large macropores, which resulted from the release and removal of CO_2_ during the droplet’s drying process. The presence of complex inner structures elucidates the mechanism behind the accelerated interaction with exudates and the subsequent acceleration in the in situ gel formation process.

**Figure 5:**
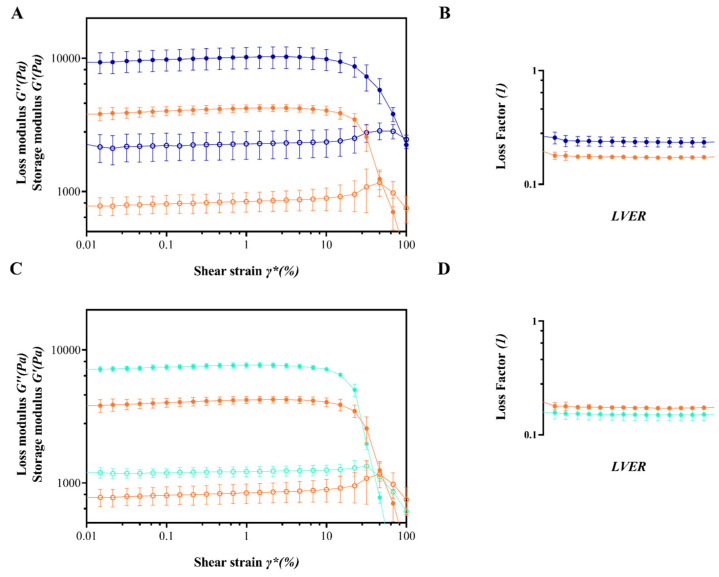
Comparison of rheological properties between APC 117 (orange line), APC 117-5Et (blue line), APC 117 (orange line), and APC 117-10Bic (green line). Panels (**A**,**C**): loss (○) and storage (●) moduli as a function of shear strain; panels (**B**,**D**): loss factor behavior in the linear viscoelastic range.

In order to assess the influence of co-solvent utilization or the incorporation of functional excipients on the properties of APC hydrogels when formed in situ, a comprehensive rheological characterization was conducted. This analysis focused on the hydrogel made with powders that exhibited superior outcomes in terms of gelling rate and SWF uptake, namely APC-117-5Et and APC-117-10Bic, in comparison to the blank formulations. The results of this characterization are presented in Figure 5. The comparison of the amplitude sweep test conducted on both APC 117 and APC 117-5Et gels (Figure 5A) points out the influence of ethanol as a co-solvent on G′ and G″, resulting in a shift of the crossover point towards higher shear strain values that have a favorable impact on the viscoelastic properties of the gel. Particularly significant is the assessment of the loss factor in the linear viscoelastic range for both gels (Figure 5B). The use of ethanol as feed co-solvent led to obtaining a hydrogel characterized by a more liquid-like consistency, as underlined by the increase in the value of the loss factor from 0.17 to 0.25. It is possible to speculate that changes in the strength of the hydrogel are due to the active role of ethanol on the polymer interaction during the spray drying process, forming a more heterogeneous matrix. This phenomenon could also be related to the increased standard deviation that was observed [48,49].

By focusing attention on Figure 5C, where the comparison between the rheological properties of hydrogels from APC 117 and APC 117-10Bic is reported, it is possible to observe a distinctly different behavior in G″. Here, there is an almost negligible alteration in the curve angle shift, indicating that bicarbonate incorporation had a positive impact on the stiffness of the gel matrix. This finding is in alignment with the assessment of gel consistency (Figure 5D), supporting an enhancement of the gel structural integrity due to the addition of bicarbonate in the feed (Figure 5D).

**Figure 6:**
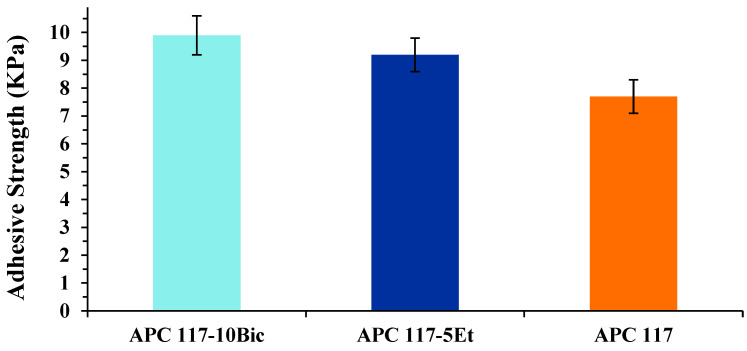
Adhesive strength of hydrogels made on APC 117-5Et and APC 117-10Bic in comparison with blank APC 117 over time.

To corroborate this hypothesis, adhesive strength analyses of the hydrogel formed in situ were evaluated using a modified protocol of ASTM D3808 standard, with a SWF-conditioned nitrocellulose membrane filter as the adhesion substrate. As shown in Figure 6, the adhesive strength of the formulations was in the range of 7.7–9.9 kPa, with the highest adhesiveness exhibited by APC 117-10Bic and APC 117-5Et (9.2 KPa). Interestingly, APC 117-10Bic was able to provide a stronger initial adhesiveness, probably due to its increased gel stiffness, than APC 117-5Et, which has demonstrated the ability to conform more effectively to surfaces, probably due to its liquid-like consistency. This range of adhesiveness levels can facilitate the gentle and atraumatic removal of the gel from the wound bed after use while also minimizing the risk of unintended removal.

**Figure 7:**
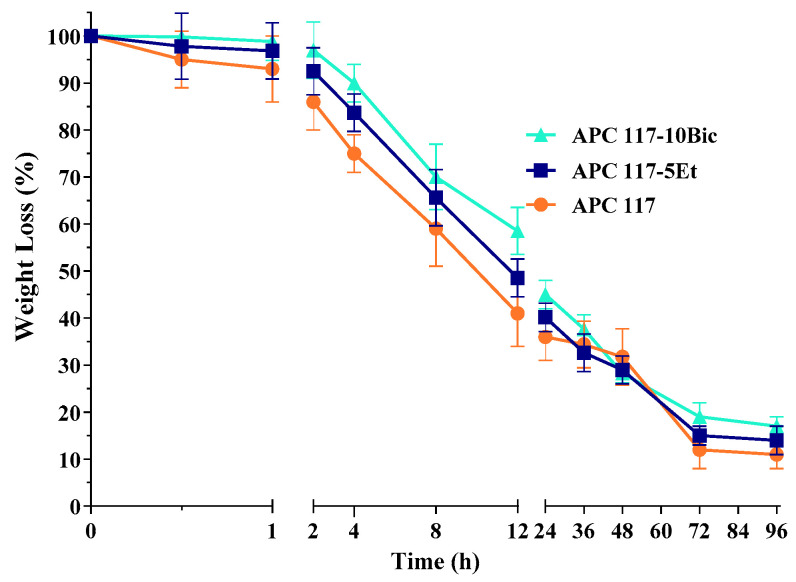
Weight loss from hydrogels made on APC 117-5Et and APC 117-10Bic in comparison with blank formulation APC 117 over time.

In Figure 7, the hydrogel water loss behavior after the complete SWF uptake is reported. Such data are able to provide insights about the possibility of the hydrogel becoming dry after the exudate efflux from the wound has stopped [50]. After 1 h, the gels formed in situ exhibited a minimum decrease in water uptake, between 7% and 2% for APC 117 and APC 117-10Bic, respectively, probably due to a higher strength of polymeric entanglement that led to a reduction in free water mobility, as suggested from the rheological results. Moreover, fluid loss in the first 12 h can be observed, with a higher value for APC 117 (60%) and lower for APC 117-10Bic (40%), while after 48 h hydrogels still retained over 30% of the fluid. Subsequently, there was a tiny amount of water loss from the gel until equilibrium was reached using APC 117, which retained 10% of the water, APC 117-5Et, which retained 13%, and APC 117-10Bic, which retained 16%. In summary, APC 117-10Bic showed a lower water loss rate in comparison to the other hydrogels due to its capability to uptake a great amount of fluid. This profile could be advantageous for dressing wounds with more exudates, such as chronic wounds [51].

Water vapor transmission rate (WVTR) was calculated to ensure the ability of the dressing to maintain an adequate level of moisture at the wound bed. A low value of WVTR leads to an accumulation of exudate on the wound surface and consequently prolonged healing time and possible promotion of infections, whereas high values can lead to dehydration of the wound and traumatic removal of the dressing. Therefore, appropriate WVTR with values ranging between 80–105 g/m^2^/h is recommended to control the level of moisture at the wound bed [52,53]. APC 117 gel formed in situ exhibited a WVTR of 97 g/m^2^/h. As expected, due to the consistency of the gels highlighted in the rheological studies, both APC 117-5Et and APC 117-10Bic had slightly reduced WWTR values, 95 and 92 g/m^2^/h, respectively.

**Figure 8:**
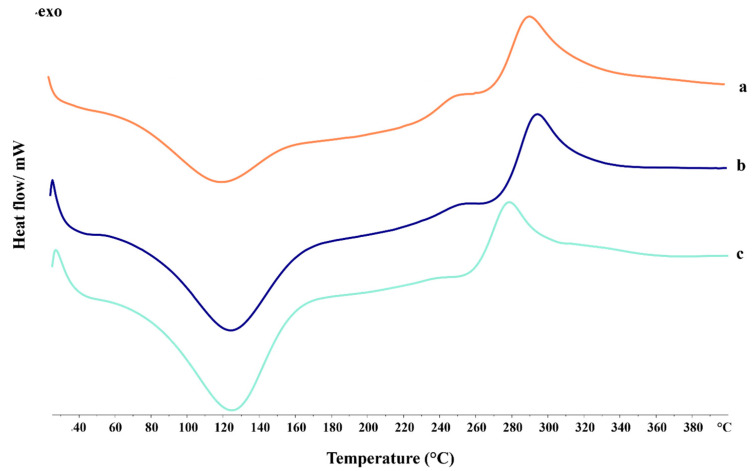
DSC thermograms of APC 117 (**a**), APC 117-5Et (**b**), and APC 117-10Bic (**c**).

Differential scanning calorimetry (DSC) was used to study the thermal transitions of materials. All formulations (Figure 8) showed a broad endothermic peak around 120 °C corresponding to water removal [54]. Focusing on this phenomenon, the value of the Δ energy (mJ) of APC 117-10Bic exceeded that of the blank formulation by approximately 78%, indicative of an increased energy requirement, possibly attributed to the partial degradation of bicarbonate [55]. Moreover, an exothermic peak emerged at approximately 300 °C for all formulations, which was related to the decomposition of chitosan [56]. The shift of this peak to a lower temperature in APC 117-10Bic can potentially be attributed to bicarbonate degradation, leading to a diminution in the physicochemical stability of the powders [57]. On the contrary, APC 117-5Et did not show any significant differences compared to APC 117, suggesting that the use of ethanol as a co-solvent did not induce significant alterations in the thermal behavior of the formulation.

**Figure 9:**
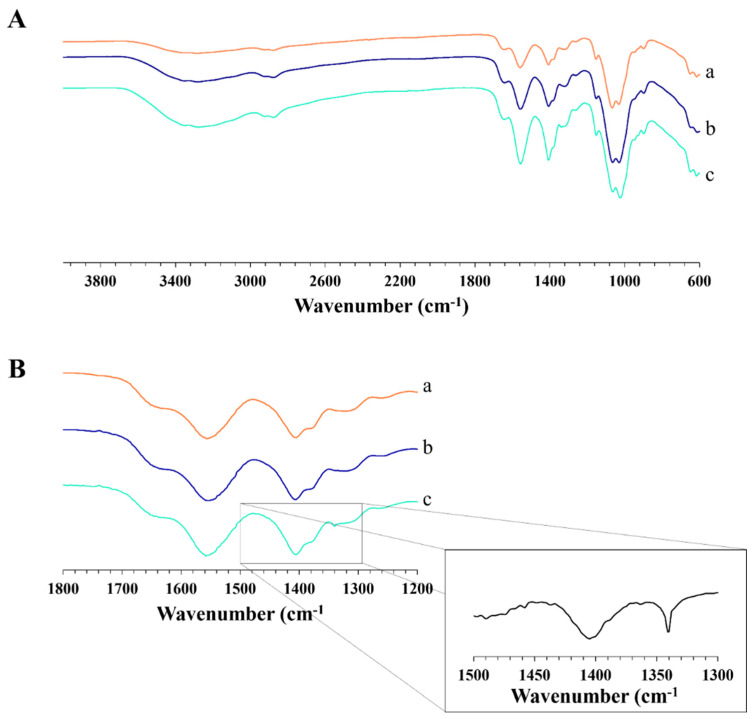
FT-IR spectra of APC 117(a), APC 117-5Et (b), and APC 117-10Bic (c). Panel (**A**): spectra in the wavelength range from 4000 to 600 cm^−1^; panel (**B**): spectra in the wavelength range from 1800 to 1200 cm^−1^. The curve reported in the square represents the result of the mathematical subtraction of the APC 117 spectrum from APC 117-10Bic.

The infrared spectrum was used to analyze the formation of intermolecular interactions. APC 117 presented different bands related to the single polymers (Figure 9A). Specifically, the bands at 1603 cm^−1^ and 1400 cm^−1^ are associated with the antisymmetric and symmetric stretching vibration of the carboxyl group of the alginate [58], while at 1549 cm^−1^, the bending vibration originates from the primary amine group, overlapping with the amide II vibration of chitosan. Furthermore, an indicative band linked to the C-O-C asymmetric stretching of chitosan emerged at 1145 cm^−1^, while the stretches of C-O for CH_2_-OH and CH-OH were evident at 1059 cm^−1^ and 1023 cm^−1^, respectively [59]. The presence of a broad OH absorption band between 3000–3600 cm^−1^ obscured the characteristic signal of the amino group [60]. The presence of ethanol in APC-5Et is not detectable, and this may indicate that the addition of the co-solvent did not induce any chemical alteration within the polymer matrix during the formation of the particles.

Further investigation in a narrowed wavelength range pointed out the residual presence of bicarbonate within the powder (Figure 9B). Specifically, in APC-10Bic, a distinctive symmetric stretching of COO–, attributed to its interaction with free water, emerged at 1340 cm^−1^ [61]. This hypothesis was also confirmed by the evaluation of the pH of the gel which revealed an increase from 6.00 in the case of APC 117 to 6.30 for APC 117-10Bic. In fact, the reaction between HCO_3_^−^ and H^+^ present in the medium of the solution produced carbon dioxide (CO2), thus increasing the pH value concerning blank formulation [62].

## 3. Conclusions

This study aimed to produce APC polymeric particles using mini spray drying for wound-healing applications. The microparticles were designed to move quickly into a hydrogel with enhanced gelling and adhesion properties when in contact with wound exudates. The effects of different co-solvents (ethanol and isopropanol) and salts (sodium bicarbonate and ammonium carbonate) on the process yield, particle size and morphology fluid uptake, gelation time, rheology, and adhesion were investigated. The results showed that the microparticles with 5% ethanol and 10% sodium bicarbonate had the best performance, with rapid gelling (less than 30 s) and high fluid uptake (about 10 times their weight in water). The rheological and adhesion tests revealed that these microparticles had optimal adhesiveness levels (7.7–9.9 kPa) that could facilitate easy and painless removal after use, as well as minimize the risk of accidental detachment. The microscopic analysis demonstrated the formation of porous inner structures that influenced the fluid uptake, gel formation, and transpiration of the microparticles. The study also provided insights into the mechanisms behind the particle formation and the gelation process, which were related to the complex formation of alginate–chitosan polyelectrolytes, the evaporation rates, and the diffusion coefficients of the solutes. Overall, the study provides insights into optimizing the hydrogel formed in situ and intended for use in wound care, using in vitro data to highlight the possibility of tailoring the microparticulate powder’s properties in order to accommodate chronic wound treatment needs.

## 4. Materials and Methods

Sodium alginate from brown algae (1% viscosity 35 mPa s; mannuronic/guluronic ratio 70/30) was kindly donated by Dompè S.p.A (L’Aquila, Italy), whereas pectin amid CF 025 D (amidated low methoxyl grade, degree of esterification 23–28%, degree of amidation 22–25%, molecular weight 120 kDa) was kindly donated by Herbstreith & Fox (Werder/Havel, Germany). Chitosan low molecular weight (50,000–190,000 Da,1% viscosity in acetic acid 20–80 mPa s; 75–85% deacetylated) was purchased from Sigma Aldrich (Milan, Italy). Sodium bicarbonate, ammonium carbonate, sodium chloride, as well as acetic acid, isopropanol, and acetone were acquired from Sigma Aldrich (Milan, Italy). Absolute ethanol was obtained from VWR Chemicals (VWR International, Radnor, PA, USA). Mycological peptone was purchased from Oxoid Ltd., Basingstoke, Hants, UK) and fetal bovine serum that was qualified and heat-inactivated was obtained from Gibco (Thermo Fischer Scientific, São Paulo, Brazil). All other chemicals used in this work were of commercial analytical grade.

### 4.1. Powder Production

Alginate–pectin–chitosan (APC) powders were produced using mini spray drying, setting the total concentration of the polymers at 0.15% (*w*/*v*) and the polymers ratio at 1:1:7 (APC 117). To the blank formulation APC 117, various co-solvents and functional excipients able to facilitate the elimination of water during spray drying and obtain particles with lower density and greater porosity, capable of further facilitating interaction with wound exudates, have been added.

#### 4.1.1. Alginate, Pectin, and Chitosan Aqueous Feed

The feed solution used to obtain microparticles through mini spray drying was prepared as follows:

- Chitosan was dissolved in an acidic aqueous solution (1% *w*/*w* CH_3_COOH), at room temperature, under a gentle stirrer overnight.
- Pectin was dispersed in an aqueous solution where it was solubilized using alginate, at room temperature, under a stirrer producing the alginate-pectin (AP) solution.

After that, the two solutions were mixed through the use of Ultra-Turrax^®^ T25 (IKAWorks GmbH & Co., Staufen, Germany), thus obtaining the final feed (APC) to process by spray drying.

#### 4.1.2. Alginate, Pectin, and Chitosan with Co-Solvent Feed

To prepare the APC feed solution with different percentages of co-solvent, various mixtures of ethanol, and ethanol/acetone in a 1:1 ratio or isopropanol, were considered. Subsequently, each of them, as well as the AP solution, were added to the chitosan solution before the formation of the final feed. Afterward, all the components were homogenized by Ultra-Turrax.

#### 4.1.3. Alginate, Pectin, and Chitosan with Functional Excipients

The addition of excipients, sodium bicarbonate or ammonium carbonate in different concentrations, happened directly in the APC solution under a gentle stirrer.

#### 4.1.4. Microencapsulation Process

All feeds were processed by a Mini Spray Dryer B-290 (BuchiLaboratoriums-Tecnik, Flawil, Switzerland) with optimized parameters: aspirator 100%, drying airflow 560–580 L/h, air pressure 6 atmospheres, feed rate 3.5 mL/min, 120 °C inlet temperature, 65–68 °C outlet temperature, and nozzle diameter 0.5 mm. At the end of each process, the powder was collected and kept in closed vials and successively analyzed in terms of yield, calculated as the ratio between the amount of product obtained and the total amount of the material processed.

### 4.2. Morphology and Particle Size Distribution

The morphology of all powders was observed by scanning electron microscopy (SEM), using a Tescan Solaris instrument (Tescan Orsay Holding, Brno, Czech Republic). A minimum quantity of each sample, according to the standard ISO 13322-1, was deposited on an aluminum stub (Agar Scientific, Stansted, UK) and coated with a thick gold layer (200–400 Å) (LEICA EMSCD005 metallizator).

The Focused Ion Beam technique, coupled with SEM microscopy (FIB-SEM), was used to obtain images of the internal morphology of the powders from cross sections of them. Milling (50 pA, 30 kV) was carried out without gallium deposition. SEM imaging was then conducted at 55° tilt using a 5 keV electron beam.

Mean diameter was calculated directly from SEM images using ImageJ, a Java-based public domain image-processing and analysis program, developed by the National Institutes of Health (NIH), Bethesda, MD, USA [63].

### 4.3. Fluid Uptake Ability

Fluid uptake experiments were conducted using the Franz diffusion cell. Briefly, about 5 mg of powder was positioned on the membrane disc filter (nitrocellulose HVLP, Merck Millipore, 45 µm), previously weighed, and collocated on the vertical receptor compartment of the Franz cell. It was filled with SWF and thermostated at 37 °C. It was composed of 50% fetal calf serum and 50% maximum recovery diluent consisting of 0.1% (*w*/*v*) peptone, a peptic digest of animal tissue, and 0.9% (*w*/*v*) sodium chloride [64,65]. At regular time intervals, the weight of the swollen powder was measured to observe a constant weight. The degree of swelling was calculated according to the following formula:

(1) $$Fluid\;uptake=\frac{Wt}{W0}\times 100$$

where *Wt* is the weight of the powder soaked in fluid at time *t* and *W*0 is the weight of the dry powder.

All experiments were performed in triplicate.

### 4.4. Rheological Studies

The rheological properties of the hydrogel generated in situ were evaluated using an Anton Paar MCR-102 Rheometer fitted with plate–plate geometry (PP25 with a diameter of 24.985 mm), selecting 1.5 mm as the measuring gap value. All formulations were subjected to amplitude sweep tests to obtain information about viscoelastic properties. Test was performed by setting the strain amplitude in the range 0.01–100% with a constant angular frequency at ω = 10 rad/s, identifying also the linear viscoelastic region. Each powder (150 mg) was placed directly on the plate, previously heated at 37 °C, and treated with SWF to form a gel. All experiments were performed in triplicate. The results of the amplitude sweep test are reported in the form of a graph as mean and standard deviation of loss and storage moduli vs. shear strain, with a particular focus on the loss factor behavior (tanδ) in the linear viscoelastic region. Generally, in practical applications, 0.01 < tanδ < 1 explains the gel-like behavior due to a balance between solid and liquid components, where 0.5 represents the transition point from a prevalent liquid-like to a prevalent solid-like state.

### 4.5. Adhesion Strength Measurement

The adhesive properties of the hydrogel formed in situ were assessed using a modified protocol of the ASTM D3808 standard with a tensile stress tester, specifically, the Electroforce 3200 testing instrument (Bose, Eden Prairie, MN, USA), following procedures detailed elsewhere [66]. Briefly, it was applied onto a nitrocellulose membrane filter (with a pore size of 0.45 µm and an area of 3.14 cm²) that had been pre-conditioned with SWF. After the gel had formed as a result of contact between the formulation and the fluid, the membrane was positioned on the movable horizontal sample holder of the apparatus. The sample holder was then moved at a constant rate of 1 mm/min, leading to the compression of the gel against the measuring head of a calibrated load cell. The force–time curve generated during this process was recorded on a personal computer, and, using LabChart 8 (AdInstruments, Oxford, UK), the force at which the gel detached from the substrate was calculated. All experiments were performed at least in triplicate.

### 4.6. Water Vapor Transmission Rate and Rate of Water Evaporation from the Hydrogels Formed In Situ

Water vapor transmission rate (WVTR) measurements were performed as described by ASTM standard (ASTM Standard, 2010). Briefly, a 25 mm hydrogel disk, formed from each powder accurately weighed and normalized concerning the amount of blank formulation when in contact with SWF, was mounted on the top of a plastic tube filled with 20 mL of distilled water.

Tetrafluoroethylene was then used to cover the edge of the hydrogel disks to avoid boundary loss. This assembly was placed in an incubator at 37 ± 0.5 °C with a humidity of 32 ± 0.2%. At defined time points, the decrease in weight was measured by a microbalance. The *WVTR* values were calculated from the slope of the weight change against time using the exposed surface area of the sample, and the equation shown in:

(2) $$WWTR=\frac{Slope}{A}$$

where *A* is the area of the sample in m^2^. The experiments were performed in duplicate.

The water evaporation rate of the hydrogel formed in situ was obtained as loss of weight over time by using the same procedure described above. After regular intervals, the weight was recorded and the one that remained was calculated by the following equation:

(3) $$Weight\;loss=\frac{W0}{Wt}\times 100$$

where *Wt* is the weight of the hydrogel at time *t* and *W*0 is the weight of the hydrogel at the beginning of the experiment.

### 4.7. Differential Scanning Calorimetry (DSC)

Differential Scanning Calorimetry (Mettler Toledo DSC 822e module controlled by Mettler Star E software, Columbus, OH, USA) was used to determine the thermal characteristics of raw materials and formulations. Next, 5–6 mg of the powder sample was weighed with a microbalance (MTS Mettler Toledo, OH, USA) before being placed in a perforated aluminum pan (40 µL). Samples, so prepared, were heated from 25 °C to 400 °C at a rate of 25 °C/min and the characteristic peaks were recorded. All DSC analysis was performed in a 150 mL/min nitrogen atmosphere.

### 4.8. Fourier Transform Infrared Spectroscopy (FT-IR)

The different produced powders were subjected to solid state FT-IR characterization in the range of 4000–600 cm^−1^ using Spotlight 400N (FT-NIR Imaging System, Perkin Elmer Inc., Shelton, CT, USA) equipped with a MIRacle ATR accessory with ZnSe crystal plate. The spectra were obtained at a resolution of 2.0 cm^−1^ using 128 scans. To better identify differences between samples, a short analysis was performed in a range from 1800 to 1200 cm^−1^, increasing the number of scans to 254. The results were evaluated using the different spectrum functions of the software Spectrum. Concerning the detailed analysis, a mathematical difference of normalized APC spectrum from normalized APCH spectrum was reported (to normalize spectra, an auto calculation of the factor to obtain the least square difference spectrum was used).

## Figures and Tables

**Table 1:** Composition, process yield, and particle size of powders produced by mini spray drying obtained by aqueous feed with different co-solvents or the addition of salt as functional excipients.

Sample	H_2_O—Co-Solvent Ratio	Salt Concentration (*w*/*w*)	Process Yield (%)	Mean Diameter (µm) ± SD
APC 117	100:00	-	61.5	3.65 ± 0.01
APC 117-5Et	95:5 Ethanol	-	55.8	5.05 ± 0.06
APC 117-10Et	90:10 Ethanol	-	55.8	4.09 ± 0.02
APC 117-20Et	80:20 Ethanol	-	45.7	4.80 ± 0.19
APC 117-20EtA	80:10:10 Ethanol/Acetone	-	55.6	2.91 ± 0.01
APC 117-5Iso	95:5 Isopropanol	-	50.3	3.27 ± 0.05
APC 117-10Bic	100:00	10% Sodium Bicarbonate	64.1	2.89 ± 0.11
APC 117-10Car	100:00	10% Ammonium Carbonate	59.3	2.11 ± 0.09

## Data Availability

The data presented in this study are openly available in article.
